# Acute aortic dissection in a young patient without Marfan fibrillinopathy: a case report

**DOI:** 10.1186/1757-1626-2-7076

**Published:** 2009-04-28

**Authors:** Panagiotis Hountis, Panagiotis Dedeilias, Konstadinos Bolos

**Affiliations:** 1 Department of Cardiac Surgery, Evaggelismos Hospital, Ipsilantou 45-47, Athens Greece

## Abstract

**Introduction:**

Acute aortic dissection is a rare clinical entity that mainly affects patients older than 50 years. It is unusual in younger patients and its presence has been traditionally associated with trauma, Marfan syndrome, bicuspid aortic valve and pregnancy. Heavy weightlifting and other fibrillinopathies have been also implicated in the literature. We present here the case of a 26 year old male with acute aortic dissection type A (De Bakey II), without family history of connective tissue diseases and signs of Marfan syndrome.

**Case presentation:**

The patient is a 26-year-old Caucasian Greek male who was presented in the emergency department with acute chest pain. Computerized tomography with contrast material showed the presence of an ascending aortic aneurysm with the question of an acute dissection type A (De Bakey II). The patient was emergently operated with replacement of the affected aortic segment and he had an uneventful clinical course. Three years follow up is essentially normal.

**Conclusion:**

Although extremely rare, aortic dissection is always a possibility in the differential diagnosis of chest and/or back pain in young patients and should be thoroughly investigated. The presence of an associated aneurysm makes the possibility even higher. The disease, if undiagnosed, carries the same mortality rates as in the older population. Prompt surgical intervention offers a possible cure and long term survival benefit for the patients.

## Introduction

Type A aortic dissection (AD), is the presence of dissection proximal to the left subclavian artery (Stanford classification). According to De Bakey classification, type II is the presence of the dissected area confined on the ascending aorta. Aortic dissection is considered surgical emergency and it has been well documented, if untreated the mortality rate is extremely high. It has been estimated that mortality approaches 1% per hour for the first 48 hours and exceeds 80% during the first month [1].

The pathogenesis of AD has been attributed to a tear in the intima (primary tear), which allows the blood to flow into the aortic wall media creating the false lumen. Another possible mechanism that has been proposed is the rupture of vasa vasorum and the creation of an intramural hematoma. This hematoma results in increased wall stress and intimal disruption [2]. This type of dissection must be operated emergently and the extent of the reconstruction is dictated by the presence of aortic valve insufficiency and the proximity of the dissection to the aortic arch.

## Case presentation

The patient was a 26-year-old Caucasian Greek male (178 cm, 75 kgs) was admitted to the Cardiology Department for the evaluation of a retrosternal constant pain of 3 hours duration. The pain started abruptly without any preceding symptoms. The pain was excruciating retrosternal pain and radiating to his right arm with a 8/10 intensity. The examination of the patient was unremarkable and his medical, surgical and family history was negative. The patient had no clinical features of Marfan syndrome. His biochemical values were normal.

**Figure 1:**
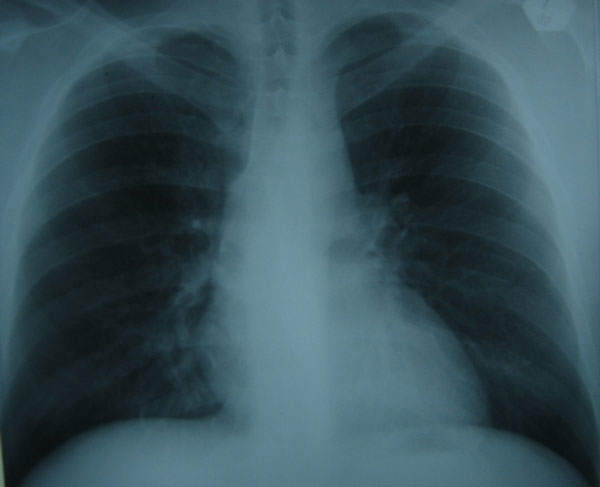
**Posterolateral Chest radiography of the patient was essentially normal**.

**Figure 2:**
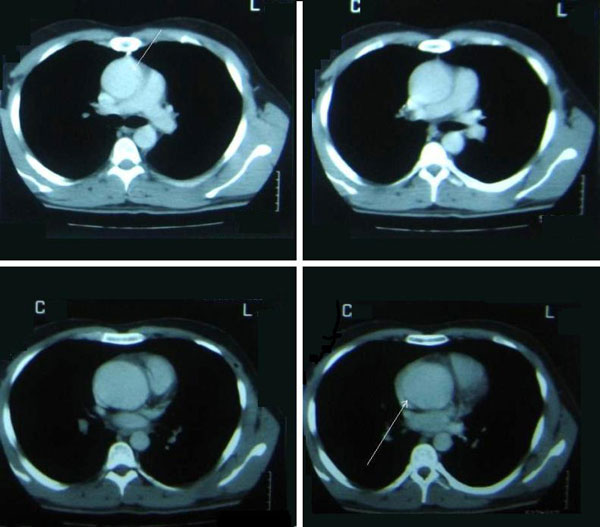
**Computerized Tomography of the Chest showed the aneurysm of the ascending aorta and was highly suspicious for proximal circumferential aortic dissection and distal re-entry point (arrows)**.

On examination, the patient was now in moderate pain (4-5/10) but he was very anxious and worried because he had never experienced this type of pain. He actually was in active duty in the army until recently (15 days before) and he was extremely active to all the exercises he had to perform. He never had any form of disease or hospitalization. His pulse was 100/min and his blood pressure was 130/65 mmHg. No murmurs or extra sounds were audible on cardiac examination. Chest x-ray was essentially normal (Figure 1). ECG was on sinus rhythm without any abnormalities. Cardiac ultrasound set the question of aortic dilatation with the possible presence of hematoma in the aortic wall or a small intimal flap in the proximal ascending aorta. Although there was an intact aortic and mitral valve function. No pericardial effusion was noted. A chest computerized tomography showed the presence of an ascending aortic aneurysm at 6 cm and a circumferential aortic tear with the possible presence of an intimal tear in the ascending aorta as well (Figure 2).

The patient was emergently operated. We performed ascending aortic replacement under the support of cardiopulmonary bypass and moderate hypothermia. Intraoperatively a complete circumferential aortic tear was noted just above the sinotubular junction. The entry and exit point of the dissection were confined in the ascending aorta. Ascending aorta was replaced with a 26 Dacron graft. The patient recovered well and discharged on seventh postoperative day. Pathology revealed no specific findings in the resected aortic segment. A three years follow up showed that the patient was negative and in excellent clinical condition.

## Discussion

Aortic dissection is mainly a disease of older age with a mean onset at 56.5 years. Patients younger than 19 years represented only 3.5% in two large series [3,4]. In young patients the disease has been associated with generalized connective tissue disorders. The most common etiologies are the vascular type of Ehlers-Danlos syndrome (EDS), Marfan syndrome (MS) and more recently the Loeys-Dietz syndrome (LDS) [5]. Weightlifting is another recently recognized factor with high prevalence in young otherwise healthy patients [6]. The most usual etiologic factor of aortic dissection in young patients in Marfan syndrome, related with defective fibrillin 1 synthesis. Fibrillin-1 is the lipoprotein that serves as the framework for elastin which is the major elastic component of the aortic wall.

It has to be noted that acute aortic dissection in a young patient is an extremely rare disease and the diagnosis difficult to suspect especially in cases without any other prominent symptoms or signs. In general the symptoms are atypical and involve retrosternal pain radiating to the back in a tearing fashion. In cases of dissection involving the great vessels to the brain loss of consciousness and stroke signs may be noted. In general, the signs and symptoms are related to the extend of the arterial branches that are involved in the various organs and the relative adequacy of perfusion of theses organs from the true or the false lumen. Coronary insufficiency, aortic valve insufficiency, paraparesis, bowel and renal ischemia and upper and lower extremities ischemia may arise suddently and must be thoroughtly investigated for in cases of aortic dissection. In suspicious cases, Computerized Tomography Scan with contrast material and Transesophageal Echocardiogram are considered the golden standard for the diagnosis.

In all the cases of aortic dissection involving the ascending aorta emergency surgical replacement is the only possible cure for the patient. This disease carries a high mortality rate if left untreated. Surgical reconstruction aims at resection and replacement of the ascending aorta and elimination of the risk of dissection in distal parts of the aorta with catastrophic consequences namely intrapericardial rupture, coronary artery dissection and aortic valvular insufficiency.

Although no marfanoid features were noted on examination in this patient, there is always a possibility that a form of fibrillinopathy may predisposed him to the aneurysm formation and the dissection of the aorta. His intense training as he described it during his military service along with heavy weightlifting may be another possible explanation although difficult to confirm. The presence of a congenital structural anomaly is unlikely, as he had a previous cardiac examination for entry into army and he had a full strenuous body activity for at least six months without any symptoms.

## Conclusion

This case stresses the possibility of the presence of aortic dissection even in young patients without any prior symptoms or phenotypic characteristic of connective tissue disorder. The disease may be easily misdiagnosed for other cardiac, muscular, neurological, esophageal or renal diseases. This possibility must be always kept in mind because the disease if left untreated is highly fatal. Prompt surgical intervention with replacement of the affected part of the aorta offers a survival benefit for the patient.

## List of abbreviations

AD: Aortic dissection; EDS: Ehlers-Danlos syndrome; MS: Marfan syndrome; LDS: Loeys-Dietz syndrome; ECG: Electrocardiogram.

## Consent

Written informed consent was obtained from the patient for publication of this case report and accompanying images. A copy of the written consent is available for review by the Editor-in-Chief of this journal.

## Competing interests

The author(s) declare that they have no competing interests.

## Authors' contributions

PH analyzed and interpreted the patient data, PD was a major contributor in writing the manuscript, KB was a major contributor in writing the manuscript. All authors read and approved the final manuscript.

## References

[B1] Patel Himanshu J Deeb Michael G Ascending and Arch Aorta: Pathology, Natural History, and Treatment Circulation 2008 118 188 195 10.1161/CIRCULATIONAHA.107.690933 18606928

[B2] Green GR Kron IL Franco KLVerrier ED Advanced Therapy in Cardiac Surgery 2003 BC Decker Inc 304 310

[B3] Fikar CR Koch S Etiologic factors of acute aortic dissection in children and young adults Clin Pediatr (Phila) 2000 39 71 80 10.1177/000992280003900201 10696543

[B4] Hogan CJ An aortic dissection in a young weightlifter with non-Marfan Fibrillinopathy Emerg Med J 2005 22 304 305 10.1136/emj.2003.011080 15788848 PMC1726717

[B5] Aalberts JJ van den Berg MP Bergman JE The many faces of aggressive aortic pathology: Loeys-Dietz syndrome Neth Heart J 2008 16 299 304 18827873 10.1007/BF03086168 PMC2553155

[B6] Gwan-Nulla DN Davidson WR Jr Grenko RT Aortic dissection in a weight lifter with nodular fasciitis of the aorta Ann Thorac Surg 2000 69 1931 1932 10.1016/S0003-4975(00)01210-8 10892951

